# Quantum Oscillations of the Nanoscale Structural Inhomogeneities of the Domain Wall in Magnetic Bubble

**DOI:** 10.1186/s11671-015-1175-x

**Published:** 2015-12-04

**Authors:** A. B. Shevchenko, M. Yu Barabash

**Affiliations:** G.V. Kurdymov Institute of Metal Physics, National Academy of Science of Ukraine, 36 Vernadskogo pr, 03680 Kyiv−142, Ukraine; Technical Centre, National Academy of Science of Ukraine, 13 Pokrovskya str, 04070 Kyiv, Ukraine

**Keywords:** Uniaxial magnetic film, Magnetic bubble, The domain wall, The vertical Bloch line, The Bloch point, Quantum oscillation

## Abstract

It is shown that at low temperatures, quantum oscillations of nanoscale structural inhomogeneities (the vertical Bloch line and the Bloch point) occur in the domain walls of cylindrical magnetic domains formed in a uniaxial magnetic film with strong magnetic anisotropy. The conditions for the excitation of these oscillations are determined.

## Background

The investigation of localized structural inhomogeneities of the domain walls (DWs) in ferromagnetic materials is one of the topical problems of the physics of nanoscale phenomena. In this connection, the magnetic films with a strong uniaxial anisotropy are particularly distinguished. In these, film strips and cylindrical magnetic domains (magnetic bubbles) are formed in external magnetic field, and the internal structure of the DWs is characterized by the presence of vertical Bloch lines (BLs) and Bloch points (BPs) [1]. The vertical BLs and BPs are stable nanoscale structures (≤10^2^ nm) that significantly affect the physical properties of domains. Many aspects related to the detection, monitoring, and control of these inhomogeneities have been well studied (see for example, the review [2] and references therein). The results of these researches determine the prospects of using the vertical BL and BP as hardware components in the microelectronics [3]. The further development of the BL and BP application is related to the investigation of their quantum properties that occur at low temperatures [4–8].

The external magnetic fields applied to these systems are an important factor that influences the dynamics (including quantum) of DW structural inhomogeneities. Their interaction can result in a situation when the vertical BL harmonically oscillates near the equilibrium position in the domain wall in magnetic bubble [9]. Similar effect can occur for the BP, which oscillations are caused by domain demagnetization field [10]. Meanwhile, as the vertical BL and BP are quantum objects, it is natural to raise the question about the influence of external perturbation on the excitation of quantum oscillations of these nanoscale systems. Moreover, it is appropriate to investigate this phenomenon for the structural inhomogeneities in the domain wall in magnetic bubble. The experimental implementation of such a domain is quite simple and does not require large values of stabilizing magnetic fields.

It should be noted that the quantum oscillations of vertical BL and BP have not been considered so far. Therefore, this problem is particularly topical, and its solution is important for the modern nanophysics and various practical applications.

This work is related to the determination of the conditions of excitation of quantum oscillations of vertical BL and BP in the domain walls of magnetic bubble.

## Methods

### Problem Solving and Discussion

Let us consider a vertical BL in a domain wall in magnetic bubble formed in a magnetic film of thickness *h*, provided that the quality factor *Q* (ratio of the energy of uniaxial anisotropy to magnetostatic energy) of the film substantially exceeds unity. In a Cartesian coordinate system with the origin at the center of the domain (the Z-axis directed along the anisotropy axis and the X-axis along the magnetization vector in the center of BL), the energy *W*_H,BL_ of interaction of BL with an external magnetic field ${\overrightarrow{H}}_y=-{H}_y^{(0)}{\overrightarrow{e}}_y$ (${\overrightarrow{e}}_y$ is a unit vector along the Y-axis) can be expressed as follows [1]:

1 $$ {W}_{\mathrm{H},\mathrm{B}\mathrm{L}}=2\pi \varDelta h{M}_{\mathrm{S}}{H}_y^{(0)}{y}_{\mathrm{BL}}, $$

where Δ is the thickness of DW, *M*_s_ is the saturation magnetization of the film, and *y*_BL_ is the coordinate of BL center displacement.

For the vertical BL stabilized by a constant magnetic field ${\overrightarrow{H}}_x=-{H}_x^{(0)}{\overrightarrow{e}}_x$ (${\overrightarrow{e}}_x$ is a unit vector along the X-axis), the frequency *ω*_BL_ of its harmonic oscillations near the equilibrium position is written as follows [9]:

$$ {\omega}_{\mathrm{BL}}={\omega}_{\mathrm{M}}\left(\varDelta {h}_x^{(0)}/\pi {\gamma}^2{m}_{\mathrm{BL}}r\right), $$

where *ω*_M_ = 4*πγM*_s_, ${h}_x^{(0)}={H}_x^{(0)}/8{M}_s<1$, *γ* is the gyromagnetic ratio, *m*_BL_ is the effective mass of BL, and *r* is the domain radius.

We can extract the elliptical phase of magnetic bubble without a loss of generality (another state of domain, e.g., one close to collapse, can be similarly considered). Then, according to [9], we get the following expressions for *m*_BL_ and *ω*_BL_:

2 $$ {m}_{\mathrm{BL}}=\frac{a}{6}{\gamma}^{-2}{\left[l{h}^{-1}-{S}_2(a)\right]}^{-1},\kern0.36em {\omega}_{\mathrm{BL}}=2{\omega}_{\mathrm{M}}{\left(\frac{3\varDelta {h}_x^{(0)}}{\pi {a}^2h}\left[l{h}^{-1}-{S}_2(a)\right]\right)}^{1/2}, $$

where *a* = 2*r*/*h*, *l* is the characteristic length of the film, *S*_2_(*a*) is the Thiele force function [11], [*lh*^− 1^ − *S*_2_(*a*)] < < 1.

Therefore, considering the above, we can write the energy of quantum oscillations of the vertical BL as follows:

3 $$ {E}_{\mathrm{n},\mathrm{B}\mathrm{L}}=\hslash {\omega}_{\mathrm{BL}}\left(n+1/2\right), $$

where *ℏ* is the Planck constant, and *n* = 0, 1, 2 …

We will study the oscillations of BL by means of quasi-classical approximation when *n* > > 1 (assessment of its applicability is given below). In this approximation, the amplitude of quantum oscillations *A*_n,BL_ is determined by comparison of their total mechanical energy ${m}_{\mathrm{BL}}{\omega}_{\mathrm{BL}}^2{A}_{\mathrm{n},\mathrm{B}\mathrm{L}}^2h/2$ and energy in (3):

4 $$ {A}_{\mathrm{n},\mathrm{B}\mathrm{L}}=\sqrt{\frac{\hslash \left(2n+1\right)}{m_{\mathrm{BL}}{\omega}_{\mathrm{BL}}h}} $$

It should be noted that the WKB method was used for the study of quantum dynamics of structural inhomogeneities in DW due to the nature of these systems, intermediate between macro- and quantum-mechanical objects.

As is known [12], the transitions of oscillator from the ground state into the quasi-classical zone can be induced by an external uniform force directed along the axis of oscillations. In our case, this force is produced by the constant magnetic field that causes small displacements of BL from equilibrium. Evidently, for the excitation of quantum levels with *n* > > 1, the average energy of interaction between BL and magnetic field ${\overline{W}}_{\mathrm{H},\mathrm{B}\mathrm{L}}={W}_{\mathrm{H},\mathrm{B}\mathrm{L}}/2$ should considerably exceed the “interlevel” energy of the BL oscillator *ΔE*_BL_ = *ℏω*_BL_. Therefore, equating the formulae for ${\overline{W}}_{\mathrm{H},\mathrm{B}\mathrm{L}}$ and energy of BL oscillations, and taking into account (1), we determine the average value of its center coordinates ${\overline{y}}_{\mathrm{BL}}=2\pi \varDelta {M}_{\mathrm{S}}{H}_y^{(0)}/{m}_{\mathrm{BL}}{\omega}_{\mathrm{BL}}^2$ and corresponding energy of interaction ${\overline{W}}_{\mathrm{H},\mathrm{B}\mathrm{L}}=2{\pi}^2{\varDelta}^2{M}_S^2{\left({H}_y^{(0)}\right)}^2h/{m}_{\mathrm{BL}}{\omega}_{\mathrm{BL}}^2$.

Further, substituting expressions (2) into the formula, we find the values of magnetic fields ${h}_y^{(0)}={H}_y^{(0)}/8{M}_s$ that provide the “quasi-classical” process:

5 $$ {h}_y^{(0)}>>\frac{2}{\sqrt{a}}{\left(\frac{\varDelta }{h}\right)}^{1/2}{\left({h}_x^{(0)}\right)}^{1/2}{Q}^{1/4}\sqrt{\varDelta {E}_{\mathrm{BL}}/{E}_{\mathrm{BL}}}, $$

where *E*_BL_ = 8*AQ*^− 1/2^ is the energy of static vertical BL, and *A* is the exchange constant. In this case, the probability distribution *w*_n_ of *ñ* = *W*_H,BL_/2*ℏω*_BP_ quanta over *n* discrete levels is determined by the Poisson distribution [12]:

6 $$ {w}_n=\frac{1}{n!}{e}^{-\tilde{n}}{\tilde{n}}^n. $$

Estimation of the expression (5) for the parameters corresponding to the typical magnetic bubble materials with *γ* ~ 10^7^ Oe^−1^s^−1^, *Q* ~ 10, *A* ~ 10^− 7^ erg/cm, *Δ* ~ 10^− 6^ cm, *h* ~ 10^− 4^ cm, *M*_S_ ~ (10 − 10^2^) G and domain condition *a* ~ 1, [*lh*^− 1^ − *S*_2_(*a*)]^− 1^ ~ 10^− 2^ gives

7 $$ {h}_y^{(0)}>>3.5\cdot {10}^{-5}{\left({h}_x^{(0)}\right)}^{3/4}. $$

At the same time, assuming *ñ* = 10, we find from (6) that *n* ∼ 10 is a characteristic quantum level of the oscillator excited by magnetic field, i.e., there are transitions from the ground level to upper energy levels of the oscillation spectrum of vertical BL.

Let us now consider the presence of BP in a domain wall of a magnetic bubble. In this case, the BP is affected by the normal to DW plane component of the domain demagnetization field ${H}_x^{(d)}$ which is given by the formula

$$ {H}_x^{(d)}=4{M}_S \ln \frac{z_{BP}+h/2}{h/2-{z}_{\mathrm{BP}}}, $$

where *z*_BP_ is the coordinate of the BP center displacement.

For small displacements of the Bloch point 2*z*_BP_/*h* < < 1_,_ this formula gives ${H}_x^{(d)}=16{M}_{\mathrm{S}}{z}_{\mathrm{BP}}/h$. Taking into account the obtained result, as well as the expression ${W}_{\mathrm{H},\mathrm{B}\mathrm{P}}={\pi}^2\varLambda \varDelta {z}_{\mathrm{BP}}{M}_{\mathrm{S}}{H}_x^{(1)}$ for the energy of interaction between BP and magnetic field ${\overrightarrow{H}}_x={H}_x^{(1)}{\overrightarrow{e}}_x$ [8] that cancels the energetic equivalence of the zones of vertical BL separated by the BP, we determine the BP frequency of harmonic oscillations:

8 $$ {\omega}_{\mathrm{BP}}={\omega}_{\mathrm{M}}{\left(\frac{2\varDelta }{h}\right)}^{1/2}{Q}^{1/4}. $$

Accordingly, the energy *E*_n,BP_ and amplitude *A*_n,BP_ of BP quantum oscillations can be written as follows:

9 $$ {E}_{\mathrm{n},\mathrm{B}\mathrm{P}}=\hslash {\omega}_{\mathrm{BP}}\left(n+1/2\right),\kern0.24em {A}_{\mathrm{n},\mathrm{B}\mathrm{P}}=\sqrt{\frac{\hslash \left(2n+1\right)}{m_{\mathrm{BP}}{\omega}_{\mathrm{BP}}h}} $$

where *m*_BP_ = *Δ*/*γ*^2^ is the effective mass of BP.

Using the expression for the energy *W*_H,BP_ and formula (8) similarly to the case of vertical BL, one can easily find that the quasi-classical approximation is valid for the magnetic fields ${h}_x^{(1)}={H}_x^{(1)}/8{M}_S$ which satisfy the following condition:

10 $$ {h}_x^{(1)}>>2{\left(\varDelta /\pi h\right)}^{1/2}{Q}^{1/4}{\left( \ln Q\right)}^{1/2}\sqrt{\varDelta {E}_{\mathrm{BP}}/{E}_{\mathrm{BP}}}, $$

where *ΔE*_BP_ = *ℏω*_BP_, *E*_BP_ = 4*πAΔ* ln *Q*—is the energy of static BP.

Estimation of formula (10) (using the parameters of film and domain given above) shows that

11 $$ {h}_x^{(1)}>>3\cdot {10}^{-3}. $$

It is evident that the expressions (7) and (11) are consistent with the following requirement to the values of planar magnetic fields applied to DW: ${h}_y^{(0)},{h}_x^{(1)}<1$. Otherwise, the internal structure of DW is polarized along the field direction. Furthermore, analysis of the expressions (7) and (11) shows that the quantum oscillations of vertical BL can be excited by less strong magnetic fields as compared to the oscillations of BP. This result reflects the fact that the interlevel energy *ΔE*_BP_ of the oscillation spectrum of Bloch point *ΔE*_BP_ is $\sim {\left({h}_x^{(0)}\left[l{h}^{-1}-{S}_2(a)\right]\right)}^{-1/2}\sim 10{\left({h}_x^{(0)}\right)}^{-1/2}$ times higher than the corresponding *ΔE*_BL_ value, whereby in the case of BP, stronger magnetic fields are required for the quantum transitions from the ground level into the quasi-classical zone.

## Results and Discussion

It is expected that the quantum dynamics of structural inhomogeneities in DW should reveal itself also in the corresponding gyrotropic bending of DWs caused by their motion. Let us consider this problem using the method of gyrotropic Thiele forces [13]. At first, we will examine the oscillations of vertical BL. In this case, the BL which moves with velocity ${\overrightarrow{v}}_{\mathrm{BL}}\sim {\omega}_{\mathrm{BL}}{A}_{\mathrm{n},\mathrm{B}\mathrm{L}}{\overrightarrow{e}}_y$ acts on a DW with gyroscopic force ${\overrightarrow{\mathrm{f}}}_{\mathrm{g},x}$ that has the following density:

12 $$ {\overrightarrow{\mathrm{f}}}_{\mathrm{g},x}=\frac{M_S}{\gamma }{\left[\overrightarrow{g}\times {\overrightarrow{v}}_{\mathrm{BL}}\right]}_x, $$

where $\overrightarrow{g}=- \sin \theta \left[\overrightarrow{\nabla}\theta \times \overrightarrow{\nabla}\phi \right]$ is the vector of gyrotropic link, *θ* and *φ* are the polar and azimuthal angles of magnetization vector, that determine the internal structures of DW and BL, respectively.

Evidently, the average value of work done by this external to DW force should be related to the BL kinetic energy ${m}_{\mathrm{BL}}{v}_{\mathrm{BL}}^2/2$ [14]. Given this requirement, integration of (12) and some transformations of (1)–(4) allow establishing the quantum behavior of DW gyrotropic bending:

13 $$ {q}_{\mathrm{n},\mathrm{B}\mathrm{L}}=\frac{2\sqrt{2}}{\sqrt{\pi }}\varDelta {Q}^{1/4}{\left({\gamma}^2{m}_{\mathrm{BL}}\right)}^{1/2}\sqrt{E_{\mathrm{n},\mathrm{B}\mathrm{L}}/{E}_{\mathrm{L}}} $$

The expression for the force acting on a DW from the BP which moves with velocity ${\overrightarrow{v}}_{\mathrm{BP}}\sim {\omega}_{\mathrm{BP}}{A}_{\mathrm{n},\mathrm{B}\mathrm{P}}{\overrightarrow{e}}_z$ is similar to (12). Therefore, as in the case of vertical BL, using the functional dependence of angle *φ* [9] and formulae (8), (9), and (12), we find the DW gyrotropic bend caused by the quantum oscillations of BP:

14 $$ {q}_{\mathrm{n},\mathrm{B}\mathrm{P}}=\frac{\pi }{\sqrt{2}}{Q}^{{}^{-1/2}}{\left( \ln Q\right)}^{1/2}\sqrt{E_{\mathrm{n},\mathrm{B}\mathrm{P}}/{E}_{\mathrm{BP}}}\varLambda . $$

Estimation of (13) and (14) at *n* ∼ 10 shows that ${q}_{\mathrm{n},\mathrm{B}\mathrm{L}}\sim {10}^{-4}{\left({h}_x^{(0)}\right)}^{1/4}{\left[l{h}^{-1}-{S}_2(a)\right]}^{{}^{-1/2}}\varLambda \sim {10}^{-3}{\left({h}_x^{(0)}\right)}^{1/4}$ and *q*_n,BP_ ~ 10^− 3^*Λ*. One can see that in the case of vertical BL, the DW gyrotropic bend depends not only on the field of BL stabilization, but also on the value of external bias field, that is reflected in the factor [*lh*^− 1^ − *S*_2_(*a*)] which determines the elliptical phase of magnetic bubble. At the same time, the DW distortion caused by the oscillations of BP has a local nature and is caused by the surface tension of DW. The above mentioned characteristics of *q*_n,BL_ and *q*_n,BP_ are consistent with the conclusions of Ref. [14] about the nature of DW gyrotropic bend caused by the dynamics of BL and BP. Besides, using formulae (13) and (14), one can find that at *h* → ∞ *q*_n,BL_, *q*_n,BP_ → 0, i.e., the effect of quantum oscillations of nanoscale structural inhomogeneities in DW occurs only in thin films. This is due to the fact that with transition to bulk materials, the demagnetizing field of domain tends to zero (see also expressions (2) and (8)).

Further, basing on relation *ℏω*_BL,BP_ ~ *k*_*B*_*T* (*k*_B_ is the Boltzmann constant) and using formulae (2), (8) and the above numerical parameters, we find the temperature of this process:

$$ T\sim \left({10}^{-4}-{10}^{-1}\right)\mathrm{K}. $$

The obtained values are in the same subcritical helium temperature range as the temperatures correspond to the effects of macroscopic tunneling and above-barrier reflection of vertical BL and BP [5–8]. This fact indicates the importance of taking into account the quantum oscillations of structural inhomogeneities in DW in the studies of these phenomena.

It should be noted that in our study of quantum oscillations of BL and BP, we did not take into account the influence of dissipative force ${\overrightarrow{F}}^{(r)}$. Let us estimate its impact. According to the formalism [15], the density of dissipative force ${\overrightarrow{f}}^{(r)}$ can be written as

15 $$ {f}_i^{(r)}=-\alpha {M}_S{\gamma}^{-1}\left[{\left(\overrightarrow{\nabla}\theta \right)}_i{\left(\overrightarrow{\nabla}\theta \right)}_k+{ \sin}^2\theta {\left(\overrightarrow{\nabla}\phi \right)}_i{\left(\overrightarrow{\nabla}\phi \right)}_k\right]{v}_k, $$

where $\overrightarrow{v}$ is the velocity of structural inhomogeneity in DW.

Integration of (15) gives

16 $$ {F}_{\mathrm{BL},\mathrm{y}}^{(r)}=\frac{4{M}_S\alpha h{\overset{.}{v}}_{\mathrm{BL},\mathrm{y}}}{\gamma \sqrt{Q}}. $$

Having determined the force acting on the BL from magnetic field *F*_H,BL_ = ∂*W*_H,BL_/∂*y*_BL_ and using Eqs. (1)–(4) and (16), we find that ${F}_{\mathrm{BL},\mathrm{y}}^{(r)}/{F}_{\mathrm{H},\mathrm{B}\mathrm{L}}<<1$ in fields with

$$ {h}_y^{(0)}>>\frac{\alpha }{\sqrt{\pi }}{\left({m}_{\mathrm{BL}}{\gamma}^2\right)}^{-1}{Q}^{-3/4}\sqrt{2{E}_{\mathrm{n},\mathrm{B}\mathrm{L}}/{E}_{\mathrm{BL}}}. $$

Comparison of this relation with Eq. (5) shows that the influence of dissipation on the quantum oscillations of vertical BL can be safely neglected if magnetic field ${h}_x^{(1)}$ satisfies the following condition:

$$ {h}_x^{(1)}>6\cdot {10}^{-1}{\alpha}^2. $$

Further, considering formulae (8)–(10) and (15), we find that the effect of dissipation on the quantum oscillations of BP is negligible under the following condition:

$$ \frac{\alpha }{4}{\left(\frac{\pi }{2}\right)}^{1/2}{\left(\frac{h}{\varDelta}\right)}^{1/2}{Q}^{{}^{-1/4}} \ln Q\sqrt{E_{\mathrm{n},\mathrm{B}\mathrm{P}}/\varDelta {E}_{\mathrm{BP}}}<1. $$

This expression can be rewritten as 1.3 ⋅ 10*α* < 1 using the parameters that correspond to the typical magnetic bubble materials; it is valid for magnetic films with damping factor *α* ~ 10^− 2^ − 10^− 3^.

Regarding the estimation of the influence of dissipative force on the BP dynamics, it is worthwhile to note that in [16, 17], authors experimentally found that at room temperature, the motion of BP in yttrium iron garnets (YIG) is accompanied by high viscosity. Taking into account the relaxation terms of exchange nature in the Landau-Lifshitz equation for magnetization of a ferromagnetic [17, 18] it was explained this effect in [19]. Comparison of the results obtained by phenomenological approach with the calculated data for domain wall, motion inhibition in YIG shows that the contributions of various processes are proportional to the self-relaxation constants [20]. These constants decrease with lowering temperature. Therefore, it is expected that at low temperatures, below the magnon activation energy, the contribution of these processes is negligible, and the standard approach can be used for YIG films, which is based on the Hilbert relaxation term; we used this approach for the estimation of dissipative force acting on BP.

In conclusion, it is worthwhile to note that the effect of quantization of DW gyrotropic bend, found in the present work, allows us to propose the system comprised of the ground and activated by magnetic field levels of the BL (BP) oscillation spectrum as a basic q-bit for data recording. Thus, not only vertical BL or BP, but also the peculiarities of DW displacements determined by their quantum oscillations can be used as elements of logical algebra in the data storage devices based on magnetic materials.

## Conclusions

The possibility of quantum oscillations of nanoscale structural inhomogeneities in a domain wall, namely vertical Bloch line and Bloch point, in magnetic films with a strong uniaxial magnetic anisotropy is shown.

The quantum nature of domain wall gyrotropic bending caused by low-temperature harmonic oscillations of vertical Bloch line and Bloch point is established. This result can be used for the future developments in data quantum recording.
